# The Importance of Nutriepigenetics in Older Adults with Inflammatory Bowel Disease

**DOI:** 10.3390/nu17040620

**Published:** 2025-02-09

**Authors:** Ana-Gabriela Prada, Costina Daniela Gîță, Sandra Monica Gîdei, Doina Istratescu, Tudor Stroie, Sorina Maria Aurelian, Gabriel Ioan Prada, Ovidiu Nicolae Penes, Anca Lucia Pop, Mihai Mircea Diculescu

**Affiliations:** 1 Faculty of Medicine, “Carol Davila” University of Medicine and Pharmacy, 050474 Bucharest, Romania; ana-gabriela.prada@drd.umfcd.ro (A.-G.P.); costina-daniela.gita@drd.umfcd.ro (C.D.G.); mmdiculescu@yahoo.com (M.M.D.); 2 Department of Geriatrics, Hospital of Chronic Diseases “Sf. Luca”, 041915 Bucharest, Romania; 3 Department of Gastroenterology and Hepatology, Fundeni Clinical Institute, 022328 Bucharest, Romania; 4 Department of Geriatrics and Gerontology, “Ana Aslan” National Institute of Gerontology and Geriatrics, 011241 Bucharest, Romania

**Keywords:** nutriepigenetics, older adult, inflammatory bowel disease, organic diet, geographical distribution

## Abstract

Background: Nutriepigenetics, the study of nutritional effects on gene expression, offers new insights into the clinical variability and relapse frequency of inflammatory bowel disease (IBD). As older adult populations are frequently unrepresented in the literature regarding their nutrition’s influence on their gene expression, we considered that studying this aspect would shed light on this population group. Materials and Methods: We conducted a prospective observational study on 60 IBD patients, categorized by age, residence, and nutrition access. Patients were grouped according to age groups (“young”-old—65–74 years old and old-old—75–86 years old), gender, IBD type, organic diet preference, Simple Clinical Colitis Activity Index (SCCAI), and Harvey-Bradshaw Index (HBI) at diagnosis and after diet change. All patients were followed up at the Fundeni Clinical Institute in Bucharest, Romania, and received personalized dietary recommendations, in accordance with the European guidelines. Results: Our findings revealed that a diet that was predominantly organic had a positive impact on decreasing the number of relapse episodes, irrespective of IBD type. Predominant diets, rich in dairy and saturated fats, mostly from non-organic sources, exacerbated inflammation and increased the frequencies of relapses. Conclusions: Older adult patients who adopted a predominantly organic diet had a reduction in frequency of relapse episodes, thus proving that diet is an important part of the treatment regimen of this population.

## 1. Introduction

Inflammatory bowel disease (IBD), comprising Crohn’s disease (CD) and ulcerative colitis (UC), exhibits diverse histological and clinical presentations [1]. Older adults with IBD often present atypically compared to young adults, with silent symptoms that complicate timely diagnosis [2].

Studies indicate that Romanian older adults show a higher prevalence of UC compared to CD, influenced by genetic predispositions and environmental factors, including smoking, alcohol use, and traditional diets rich in saturated fats and dairy, which are known to exacerbate inflammation [3].

There are some genetic as well as environmental factors that influence the higher prevalence of UC. The high prevalence of smoking (34% of the general adult population) [4], as well as access to organic (3% of the cultivated area in Romania) [5] versus non-organic produce (97% of the cultivated area), have a great impact on the evolution of IBD, especially in older people. Assessing dietary habits and geographic influences is critical for better management of IBD in older adults.

One study conducted as part of the project “The geographical study of man–environment relationships in metropolitan areas” [6] suggests that in Romania, there are regional disparities in organic agriculture and region-dependent clusters of organic producers of different raw materials: one that is livestock-dominated and located in the mountain–plateau–hill region in the central, north, and northeast of Romania, and one that is crop-dominated in the west, south, and southeast. Even though there is a geographical distribution of these products, there is no consistent pattern for organic farming, due to the variabilities in local weather conditions; thus, we could not conduct a geographical distribution of nutritional patterns in Romania.

Dietary preferences and changes can have a great impact on the quality of life of older adults, especially in association with IBD, due to the sometimes absolute restrictions put in place in response to symptoms provoked by certain foods. A recent update from the American Gastroenterology Association highlighted the Mediterranean diet’s benefits in reducing relapses and inflammatory biomarkers after six months, though evidence on gluten-free and FODMAP diets remains limited [7]. There are not enough studies to prove the usefulness of a gluten-free diet in patients with IBD if there is no gluten sensitivity confirmed. Another type of diet that was studied in these patients was the FODMAP diet, which consists of a low-fermentable oligo-, di-, and monosaccharide and polyol diet, proved beneficial for symptom relief in active periods of IBD, although there was a prospective, randomized trial that found that there might be long-term side effects from this type of diet [7,8].

The International Organization for the Study of Inflammatory Bowel Diseases (IOIBD) Nutrition Cluster [9] created a guideline for specific dietary components, food groups, and food additives that may affect the nutritional requirements of patients with IBD, for better control and possible prevention of relapse episodes in CD and UC. They considered seven food groups, dietary components, and five food additives: dairy, red meat, processed meat, poultry, eggs, fruits and vegetables, fat, refined sugar, wheat and gluten, alcohol, emulsifiers, maltodextrins and artificial sweeteners, gums and thickening agents, and nanoparticles.

Studies [10,11,12] have shown through genetic testing that lactase deficiency and lactose intolerance are common among patients with IBD, which is further supported by the IOIBD Nutrition Cluster [9], and the most frequently associated disease with this type of deficiency is CD. Several epidemiological studies [7,9,13,14] have mentioned the trend of CD patients having reduced fruit and vegetable intake before disease onset, with an increase in this food group after diagnosis.

A group of researchers [15] found that nutrigenetics in IBD patients provided insights into their genetic variability in inducing an individual’s response to nutrition according to their states of health and disease, while nutrigenomics highlighted the changes in gene expression that develop because of nutrition–gene interaction. They also indicated that the genes involved in these processes were closely linked to dysregulation of an innate immune response to enteric flora, dysregulation of adaptive immune response, and a faulty epithelial barrier. These findings can guide the research on how nutrition influences IBD outcomes and disease-specific treatment efficiency by allow for the creation of a personalized nutritional prescription based on individual genetic variations. They concluded that genetic variation affects diet–gene interactions and individual nutritional requirements, which is in concordance with our study’s findings.

The place of residence of older patients is also of high importance in studying the nutriepigenetics of these patients, from the perspective of access to organic food. Rural residents are often at a diagnostic and therapeutic disadvantage, but have better access to organic products compared to urban residents, further emphasizing the role of nutriepigenetics in understanding disease progression and outcomes.

Population studies at the national level showed a multimodal distribution of people over 65 years of age predominantly in rural areas, before the COVID-19 pandemic [16,17]. After the pandemic and national quarantine period, the elderly were relocated to urban environments by people who provided their care (children, grandchildren, social workers), which led to a possible increase in social anxiety and the desire for social isolation [17,18]. On the other hand, this relocation facilitated faster access to diagnosis and treatment. These results favor a discussion related to the treatment compliance of these patients, along with a thorough investigation of the effects of social isolation on neurocognitive function, mood disorders, malnutrition, and the evolution of IBD [19,20,21].

A descriptive cross-sectional questionnaire-based study evaluating the Romanian people’s motivations for healthy eating showed that significantly more people living in urban areas were compliant with a healthy and balanced diet, while people residing in rural areas were more concerned about food properties and health attitudes [22].

Distribution by age groups helps in the study of differences and similarities between these patients, and the diagnosis and treatment of patients can be stratified with greater ease. Studies have shown that the geographical distribution of older adults with IBD plays an important role in a better stratification of the phenotype of each IBD patient [3,23,24].

We considered stratification according to place of residence, gender, and age group to better emphasize differences in nutritional habits and the influence of the type of nutrients available that contributed to gene expression and consequently to the late onset of IBD in this patient population.

Our work contributes to the nutriepigenetic research of environmental variabilities in nutrition and IBD outcomes in three ways. Firstly, based on the results from this study, we can conclude that environmental factors play an important role in nutritional effects on gene expression. Secondly, we established that there is a difference between organic and non-organic nutrition, where each has its own effects on disease outcome. Thirdly, we outlined the design implications and future research directions that emerged during this study around nutriepigenetics in older-onset IBD. Our study takes the first step in exploring and understanding this new research area. We identified the need for more research to understand the promise and the downside of the influence of nutrition in the gene expression of patients with IBD in old age.

## 2. Materials and Methods

We conducted a qualitative convergent study in a primary gastroenterology clinic in Bucharest by accessing the patients’ medical files from the hospital’s electronic database. The research methods used were qualitative and comparative analyses of the data. The initial search retrieved 1457 occurrences of patients over the age of 65. After excluding multiple presentations of the same patient, our final database included 60 patients.

The result of the initial search was screened out manually by reading the patients’ discharge papers and by discussing with the patients, to identify the eligible subjects for this topic.

We categorized them by age groups, 65–74 (“young”-old) and 75–86 (old-old); gender, female and male; place of residence, rural and urban; type of nutrition, organic and non-organic; and smoking habits, smokers and non-smokers.

Our patient population comprised all patients aged 65 and above at the moment of diagnosis. As there were no scores validated in clinical practice, as mentioned by the ECCO-ESGAR diagnostic guideline [25], we considered the Simple Clinical Colitis Activity Index (SCCAI) [26] for patients with UC and the Harvey-Bradshaw Index [27] for CD patients as valid tools for better assessment of relapse episodes at time of diagnosis and later, after diagnosis, treatment introduction and change in diet, as emphasized by Table 1. Relapse episodes were categorized into those before the diagnosis of IBD and those after the diagnosis and change in diet. We also conducted a survival analysis to check if the diet had any influence on the frequency of relapse rates.

After obtaining written consent to include the patients in this study, we contacted them via telephone to conduct an interview about their eating habits and choice of diet since being diagnosed with IBD, and we compared them to the recommendation given by their physician to exclude recall bias.

We used the mini-nutritional assessment (MNA) screening and evaluation questionnaire, and based on their answers, we obtained results regarding the types of nutrients found in their diets and the risk of malnutrition. This tool includes the types of products used in patients’ diet, i.e., the number of portions of red meat, poultry, vegetables and fruits, and milk-derived products per day, as well as the quantity of hydration per day.

Distribution by age groups and environments of origin is important for the stratification and study of patients diagnosed with IBD.

We excluded diet changes due to other comorbidities, as per the recommendation given by the IOIBD Nutrition Cluster [9] when conducting studies on the influence of nutrition on disease outcome.

Smoking habits were screened for all patients, irrespective of the type of IBD, to verify if there was any correlation between the smoking habit, gender, type of IBD and smoking habit, age group, and frequency of relapses [28]. Studies have shown that smoking has an important impact on gene expression; thus, the current study only focuses on its effects on disease outcome and if it correlated to the type of diet described.

### Data Extraction, Analysis, and Quality Assessment

Any disagreement was discussed and resolved by further analysis and careful examination. Each patient signed a consent form that was pre-approved by the ethics committee of the Fundeni Clinical Institute. Graphs were made using Excel (Microsoft Office^®^, Albuquerque, NM, USA) and patients were sorted using the Romanian Diagnostic Related Groups (DRG) codes [29]. Statistical analysis was performed by using IBM SPSS Statistics version 30.0, for conducting power analysis for partial correlation, to check for confounding variables and to obtain the statistical significance and confidence intervals of the results. We also conducted descriptive statistics to better characterize our patient population and their diet preferences (organic versus non-organic), disease outcomes, and possible effects of medication on disease outcomes. The bias correction and standard errors were zero, meaning that bootstrap resampling produced identical values across iterations, which suggests that the data are consistent and stable, with minimal sampling variability.

Eligible participants had a diagnosis of IBD, were aged 65 and older, and resided in either rural or urban areas. Exclusion criteria included patients under 65, without diagnosis of IBD, and deceased patients at the time of data retrieval. Data were analyzed using pivot tables in Excel, grouping patients by their type of IBD, age group, place of residence, MNA score, organic and non-organic produce consumption, and smoking habit.

## 3. Results

Using the results of our data analysis, we aimed to assess the influence of organic versus non-organic nutrition on IBD outcome, taking into consideration the place of residence, type of IBD, and type of produce used. The main goal was to assess how effective organic nutrition was compared to the non-organic on IBD progression in older adults.

Among the 60 patients studied, 78.33% resided in urban areas, while 21.67% were from rural regions. When studying the prevalence of IBD patients in each age group, we observed that most were in the “young”-old group (58.33%), and only 41.67% were part of the old-old group. Out of the whole data population, 33% of patients were diagnosed with CD, while 66% were diagnosed with UC.

We included in Table 2 the type of treatment used for each type of IBD in our patient lot in order to have a clearer view of the possible implications that this treatment might have had on the disease course, alongside changes in diet. Some treatment regimens, like the combination of 5-acetylsalicylic acid (5ASA) with azathioprine (AZA) and corticosteroids with mesalazine (MSZ), were not used in patients diagnosed with CD, while 5ASA as a monotherapy was used both in UC (11.67%) and CD (3.33%) patients. We did not find a correlation between the treatment used and having an organic diet. Vedolizumab (VDZ) was also used in combination with 5ASA and AZA (1.67% of UC patients) and in combination only with 5ASA for 3.33% of the UC patients. Ustekinumab (UST) was used in combination therapy in 1.67% of CD patients and in monotherapy in both the UC (8.33%) and CD (13.33%) patients, while infliximab (IFX) was used in monotherapy only in CD patients.

The rural area residents had a higher prevalence (46.15%) of organic fruit and vegetable consumption (3 or more portions of fruits and vegetables per day, as mentioned in the MNA) compared to those from urban areas (12.77%). Because patients residing in villages have access to fresh produce from their own garden, this increases the probability of them having a plant-rich diet, while “healthy” fats are also naturally found in the produce made by them. Diet variation according to seasons might also influence the disease progression and frequency of relapse episodes, but there were no seasonal data available at this time.

Upon discharge, most patients were also prescribed a personalized diet according to each patient’s unique experience with various food groups, as well as disease-oriented recommendations. Patients with UC were recommended to avoid spicy foods, condiments, and raw vegetables and fruits. The diet for CD patients was more permissive, advising them only to avoid desserts and unprocessed dairy products. Some patients (20%) had more relapses while consuming foods high in unsaturated fats, while others had more relapses after consuming milk-derived products (33%).

The most prevalent IBD was UC both in rural area (61.54%) and urban area (68.09%) residents. Out of all UC patients, 12.5% had a diet rich in organic produce, while the majority of the studied UC adults (87.5%) had less organic produce in their diets, irrespective of place of residence. Patients residing in rural areas and diagnosed with UC had a lower prevalence (40%) of non-organic produce consumption, while those residing in the urban areas had a higher consumption rate for non-organic produce (85.71%). After switching to a more plant-based diet, the latter group reported fewer episodes of relapse (2–3 per year, as compared to 5–6 per year before changing diets). At the moment of diagnosis, 75% of the patients diagnosed with UC had 17 points on the SCCAI, and a decrease of 2.5 points was observed 6 months after diagnosis, medical treatment, and change in diet in 60% of these patients. These results come to emphasize our hypothesis that people residing in the countryside have more access to fresh and organic produce.

**Figure 1:**
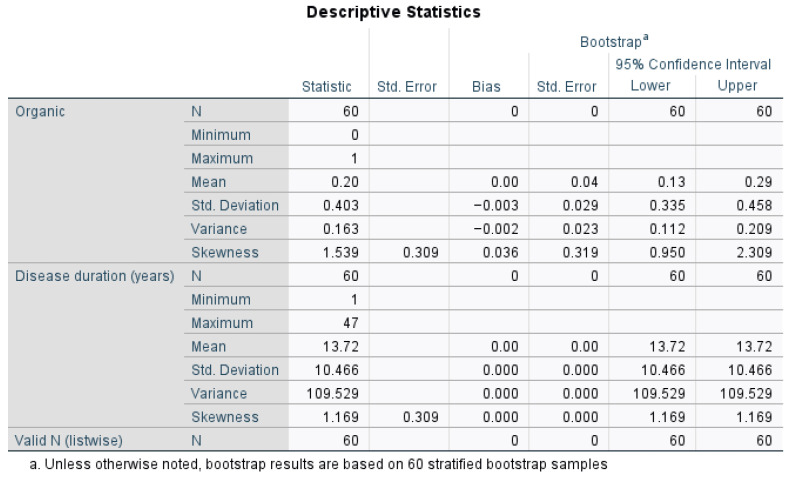
Descriptive analysis of the patient population. Std. error means “standard” error and the same for “std. deviation”, it means “standard deviation”. The N refers to the number of subjects.

As shown in Figure 1, 20% of the patients were classified as having organic diets. Their disease duration was highly variable, ranging from 1 year to 47 years, with an average of 13.72 years (Mean = 13.72 years, Standard Deviation = 10.466, Variance = 109.529, Mean CI (13.72 to 13.72)). The bootstrap confidence intervals confirmed the reliability of these statistics, especially for disease duration.

A more seasonal and organic diet was reported by patients living in rural areas (56% of the patients, approximately two portions of 50 to 100 g of vegetables per day). Patients from cities noted a less balanced diet, with less intake of vegetables (three portions of 50 to 100 g of vegetables per week), while organic produce was avoided due to higher costs. This also varied depending on the geographical distribution of patients, where patients living in a more mountainous area had more access to a high-fiber diet, less fatty foods, and more fruits.

When evaluating our patients’ nutritional status, we used MNA to screen for the risk of malnutrition, but our studies showed that there was only one patient with risk of malnutrition, which did not pose a significant statistical value.

The regression analysis of the probability of influence on relapse episodes in older adult patients with IBD when consuming predominantly organic products compared to non-organic products returned a normal probability plot. There was a moderate positive correlation between the predictors and the dependent variable (multiple R = 0.513), and 26.3% of the variability in the nutritional input (organic diet) can be explained by the frequency of IBD relapses, though our study had a high statistical significance overall (Significance F: 0.000193), as shown in Table 3.

Table 3 also shows that the predicted baseline number of relapse episodes for individuals with a non-organic diet was approximately 0.237.

The *p*-value (0.057) highlighted in Table 4 for the intercept (organic diet) indicates that the organic diet was marginally non-significant, suggesting weak evidence that the mean value of the outcome for non-organic products in their diets differed from that with organic products. The confidence interval [−0.007, 0.481] included zero, which reinforces that the outcome for non-organic eaters might not be significantly different from zero.

Both the relapse episodes at diagnosis and relapse episodes after diagnosis and change in diet significantly influenced the outcome, with the former showing a positive effect and the latter showing a stronger negative effect. This means that the organic diet had an influence on the disease outcome at diagnosis in the IBD patients, but no influence on the disease outcome after diagnosis and change in diet.

Moreover, a coefficient of 0.032 indicates that, for every one-unit increase in relapse episodes at diagnosis, the disease outcome was expected to increase by 0.032 units. This suggests that for every relapse episode at diagnosis, a change in diet could have a positive effect on the quality of life and ultimately on the gene expression in this population.

One other relevant value that enhances the validity of our study is the *p*-value of 0.031, which suggests an association between the frequency of relapse episodes at diagnosis and the incorporation of more organic produce in the diet, with a statistical significance at the 5% level (*p*-value <0.05). Thus, there is strong evidence to reject the null hypothesis, which assumed no relationship between relapse episodes at diagnosis and adopting a healthier lifestyle, by introducing organic produce in the diet.

**Figure 2:**
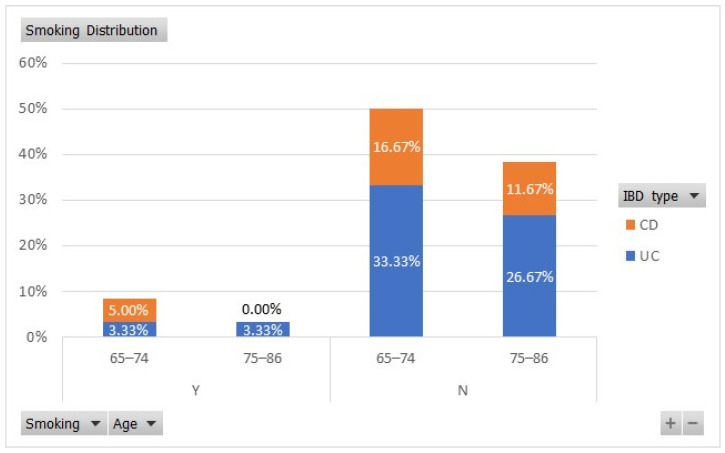
Distributions of smoking habits among older adult patients with IBD.

Smoking is another important predictor of disease outcomes in IBD patients, being a protective factor for relapses in patients with UC and an aggravating factor in those with CD [28]. We found that in our study lot, 33.33% of the 65–74-year-old UC patients were non-smokers, while 3.33% were smokers. From the old-old age group, 26.67% of patients with UC were non-smokers and 3.33% were smokers, as shown in Figure 2. In the “young”-old age group, 5% of the CD patients were smokers and 16.67% were non-smokers.

A further comparison of how smoking affected the number of relapse episodes per year at diagnosis showed that non-smokers had more flare-ups (average of 12.51 relapse episodes per year) compared to smokers (10.71 relapse episodes per year), but with no statistical significance (*p* > 0.05) due to an unequal representation of non-smokers versus smokers.

**Figure 3:**
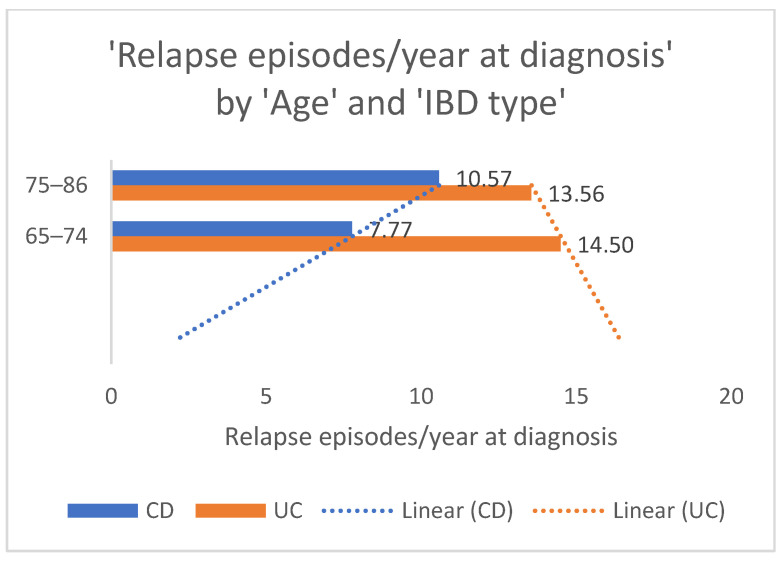
Distributions of relapse episodes per year at diagnosis according to age groups and IBD types. There is a linear forecast representation of the probable evolution of relapse episodes across age groups, both for CD and UC.

The representation of relapse episodes per year at diagnosis according to age groups is relevant for a better understanding of IBD outcomes across the older adult population. The average amount of flare-ups in the old-old patient population with CD was significantly higher than of those in the “young”-old with the same IBD, as shown in Figure 3. The linear forecast tends to show an increase in flare-ups as age advances, which can be influenced by other factors that were not the topic of the present study. There is a trend in the linear forecast for patients with UC as opposed to those with CD, with the “young”-old exhibiting more frequent relapse episodes compared to the old-old. These results can be interpreted as a probable higher inflammatory status in the younger population.

**Figure 4:**
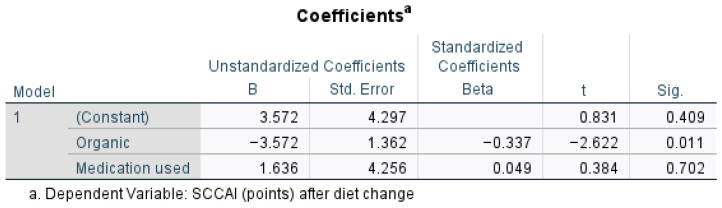
Regression analysis for outcomes of the SCCAI after diet change when medication was used and organic food was taken into account.

Figure 4 presents the regression analysis for outcomes of the scoring in UC patients after diet change, when medication was used and organic food was taken into consideration. An organic diet significantly lowers the SCCAI (*p* = 0.011), suggesting a beneficial effect, while medication use does not significantly impact SCCAI (*p* = 0.702, *p* > 0.05, t = 0.384). The strongest predictor in the model is the organic variable (Beta = −0.337), meaning the organic diet has a statistically significant impact on reducing SCCAI scores after diet change (t = −2.622, *p* = 0.011, *p* < 0.05). A one-unit increase in the organic diet score was associated with a decrease of 3.572 points in the SCCAI (B = −3.572).

**Figure 5:**
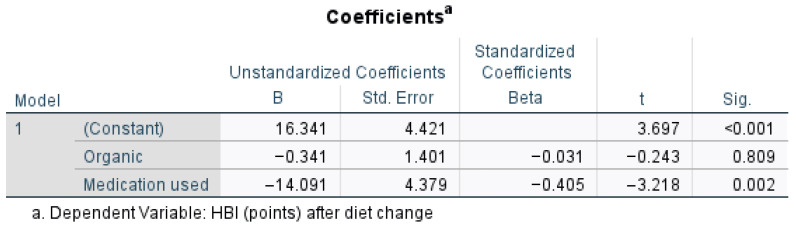
Regression analysis for outcomes related to medication use or organic food.

Medication use significantly reduced the HBI (*p* = 0.002), as highlighted by Figure 5, suggesting a strong effect, whereas an organic diet did not significantly impact the HBI (*p* = 0.809). The negative Beta for medication (−0.405) suggests it had the strongest influence on disease outcome in patients with CD. Medication use significantly predicted lower HBI scores after diet change (t = −3.218, *p* = 0.002, *p* < 0.05). A one-unit increase in the organic diet score (B = −0.341) decreased the HBI by 0.341 points, with a very small standardized effect size (Beta = −0.031). Organic diet has no meaningful impact on the HBI after diet change (t = −0.243, *p* = 0.809, *p* > 0.05).

## 4. Discussion

The present study showed that the most prevalent place of residence of our patients with IBD was in urban areas, which is comparable to the general population as highlighted by studies that took into consideration the place of residence of older adults [16,17,18]. This is of importance when taking into consideration the availability of organic products.

Our results showed that 73.7% of the variability in disease outcomes was due to factors not included in our study (e.g., random error or noise). While the change in the frequency of relapse episodes at diagnosis compared to the moment after the change in diet contributed meaningfully to explaining the type of products present in their diet, a large portion of the variation in our results is still to be researched over a longer period. Our future studies will focus on further exploring this variation.

This study highlights significant disparities in IBD diagnosis and management between urban and rural older adults. Urban patients benefitted from better access to healthcare and more varied diets, correlating with reduced inflammation and fewer relapses. Rural patients, on the other hand, faced diagnostic delays and limited dietary options, which likely contributed to disease progression.

Some studies have shown that curcumin [30,31,32] potentially has a positive effect on decreasing inflammation at the level of the large intestine. Thus, for future research, curcumin would be a nutrient of interest for our studies on decreasing the frequency of relapse episodes in older patients with IBD.

IBD leaves an important mark on the quality of life of people with this pathology, and the evaluation of their nutriepigenetics and their gerontopsychological evaluation represent important predictive factors for disease progression and therapeutic outcomes [15,33,34,35,36].

Organic produce is more accessible to patients living in rural areas compared to urban areas, thus influencing the type of diet seasonally (fruits and vegetables are mostly available during the summer, while fats are mostly consumed during the winter). Based on the responses given by the patients during the interviews we conducted within our study, the patients who lived in rural areas grew their produce in their gardens.

### 4.1. Limitations

This study analyzed only 60 patients, which is a relatively small sample size for generalizing the findings to the broader population, particularly when stratified by different diagnoses and place of residence. Additionally, the small number of rural patients (eight in total) further limited the ability to make meaningful comparisons between urban and rural populations.

Another limitation is related to the reluctance of older adults to discuss medical problems or possible new symptoms that may indicate treatment outcomes and the risk of needing surgical treatment.

Adherence to a new diet is also a habit that takes time, and research has shown that older adults prefer to keep the diet they were used to, even though it might not meet the nutritional requirements IBD patients might need [37].

### 4.2. Further Research

We will conduct cross-sectional quantitative studies in which we will also analyze other factors that can influence the early diagnosis of this type of gastrointestinal disease, as well as the ways of therapeutic management according to various specific parameters of the patients. We will also include the screening of malnutrition in older adults with IBD at each presentation at the clinic. A tailored physical activity plan proved useful in Romanian older adults to improve their hemodynamic and cognitive status [38], which is also a point of focus for our future research, as some patients mentioned sustained physical activity throughout their life.

Additionally, prospective studies investigating nutriepigenetics using targeted genetic testing of patients and their families are warranted to address diagnostic disparities and optimize dietary interventions.

Another point of discussion will be considered regarding the period patients have spent in rural areas.

Our research showed that the effects of smoking in patients are of significant relevance to the frequency of relapse episodes at diagnosis and after changing their diet, thus prompting a direction for further research over a longer period to better quantify the effects of this habit on the molecular structure of the gastrointestinal tract [37].

Screening of frailty related to malnutrition and sarcopenia will be a handy tool in older adults diagnosed with IBD, as well as in older adults without the diagnosis of IBD [39].

Medical treatment plays an important role in disease outcomes and the frequency of relapse episodes; thus, our future studies will also be focused on researching the possibility of interactions between certain types of molecules and the type of products available (organic or processed).

## 5. Conclusions

A tailored nutritional approach is essential for improving outcomes in older adults with IBD. Dietary adjustments, such as introducing more organic produce and avoiding smoking, will greatly reduce the frequency of relapse episodes in this population group. Thus, addressing disparities in healthcare access and emphasizing early diagnosis, particularly in rural settings, is crucial for effective management and treatment compliance.

## Figures and Tables

**Table 1:** Classification of IBD severity.

HBI		SCCAI	
Remission	<5 points	Clinical remission	<2 points
Mild disease	5–7 points	Active disease	>2 points
Moderate disease	8–16 points	Relapsed disease	≥5 points
Severe disease	>16 points		

**Table 2:** Distributions of treatment for each IBD type.

Treatment	UC	CD
5ASA	11.67%	3.33%
5ASA + AZA	3.33%	0%
5ASA + AZA, then VDZ	1.67%	0%
5ASA, then AZA	1.67%	0%
5ASA, then corticosteroid, then VDZ	1.67%	0%
ADA	1.67%	1.67%
Budesonide + 5ASA + UST	0%	1.67%
Corticosteroid + MSZ	1.67%	0%
IFX	0%	1.67%
MSZ	25%	3.33%
SLZ	6.67%	1.67%
UST	8.33%	13.33%
VDZ + 5ASA	3.33%	0%

**Table 3:** Regression analysis of the influence of organic versus non-organic diet on the frequency of relapses in older patients with IBD.

Regression Statistics	
Multiple R	0.513041
R Square	0.263211
Adjusted R Square	0.236897
Standard Error	0.343138
Observations	59

**Table 4:** Results of the regression analysis for the type of diet adopted by patients with IBD.

	Coefficient	Standard Error	t Stat	*p*-Value	Lower 95%	Upper 95%	Lower 95.0%	Upper 95.0%
Intercept	0.236825	0.121727	1.945543	0.056736	−0.00702	0.480674	−0.00702	0.480674
Relapse episodes at diagnosis	0.031652	0.014089	2.24653	0.028631	0.003428	0.059876	0.003428	0.059876
Relapse episodes after diagnosis and change in diet	−0.07072	0.01699	−4.1624	0.00011	−0.10475	−0.03668	−0.10475	−0.03668

## Data Availability

The data used in this study can be made available upon reasonable request due to privacy, legal and ethical reasons.
